# Implementation of a Breast Cancer Specific Multidisciplinary Tumor Board to Improve Treatment Planning and Completion

**DOI:** 10.1200/GO.22.19000

**Published:** 2022-05-05

**Authors:** Emma Joo, Matagoro Kirahi, Tara Friebel-Klingner, Leonard Washington, Anne Rositch

**Affiliations:** ^1^ Johns Hopkins Bloomberg School of Public Health, Baltimore, MD; ^2^ Bugando Medical Center, Mwanza, Tanzania

## Abstract

Bugando Medical Center (BMC) is the only tertiary cancer referral hospital in Northern Tanzania serving a population of 15 million, burdened with high breast cancer (BC) mortality rates. Multidisciplinary tumor boards (MDTB) are an evidence-based strategy shown to improve quality of treatment. Therefore, we integrated and evaluated a BC-specific MDTB as part of the standard care pathway.

**METHODS:**

A MDTB was implemented in February 2020 after working with key stakeholders and identifying a physician to lead, prepare, and guide the meetings. Once patients aged 18 and older receive appropriate diagnostics tests, results are brought to the meetings for collaborative review, resulting in a treatment planning form per patient. A standardized observational checklist was completed by a research associate at each meeting and a provider survey was conducted 3-months post-implementation to assess provider perspectives on the MDTB process.

**RESULTS:**

From February 2020 to August 2021, 630 patients with breast concerns presented to BMC. Of these, 21% (n = 131) were diagnosed with BC, of which 64% (n = 84) were discussed at tumor board and 86% (n = 72) of those discussed received a treatment plan. This occurred over 33 biweekly meetings, each averaging 1.3 hours with 14 attendees who were doctors and residents from six specialties. Survey results revealed that providers thought the MDTB improved treatment plans for BC patients (78% agree or strongly agree) and patients experienced improved outcomes (57% agree or strongly agree). Challenges included not meeting before the start of patient treatment (64%) and not having enough diagnostic information (57% often or always).

**CONCLUSION:**

The BC-specific MDTB has been in place for 20 months and has already positively impacted treatment planning and provider perspectives on breast cancer care at BMC. We will continue applying stakeholder feedback to enhance our program and focus on evaluating the completion of treatment according to the MDTB planning forms.

